# Examining the Use of Game-Based Assessments for Hiring Autistic Job Seekers

**DOI:** 10.3390/jintelligence9040053

**Published:** 2021-11-03

**Authors:** Colin Willis, Tracy Powell-Rudy, Kelsie Colley, Joshua Prasad

**Affiliations:** 1 HireVue, Inc., South Jordan, UT 84095, USA; 2 Integrate Autism Employment Advisors, New York, NY 10022, USA; tracy@integrateadvisors.org; 3 Department of Psychology, Colorado State University, Fort Collins, CO 80523, USA; kelsie.colley@colostate.edu (K.C.); joshua.prasad@colostate.edu (J.P.)

**Keywords:** autism, hiring, intelligence, selection, cognition, neurodiversity

## Abstract

Although people with autism are protected under the Americans with Disabilities Act of 1990, there is little theoretical or practical effort to determine whether traditional pre-employment assessments unfairly impact autistic job seekers. Due to the lack of emphasis on social communication, game-based assessments (GBAs) may offer a way of assessing candidate ability without disadvantaging autistic candidates. A total of 263 autistic job seekers took one of two game-based assessment packages designed to measure cognitive ability. After comparing their results to 323 college-graduate job seekers in the general population, we found that performance on the GBAs was generally similar in both populations, although some small differences were detected. Implications for hiring decisions are discussed.

## 1. Introduction

Attitudes toward who is qualified to work is undergoing a shift. Organizations today are reeling from an unprecedented labor shortage (Spiggle 2021) that, coinciding with a rapidly warming attitude toward the inclusion of “disabled” talent in the workforce, has led to an explosion of interest into tapping this large untapped labor market (Schur et al. 2014). Through the lens of neurodiversity, autistic individuals, in particular, are increasingly thought of as a skilled and reliable part of this untapped talent pool who can bring diverse perspectives and valuable traits, including a strong work ethic, heightened attention, and analytical and critical thinking skills, to an organization (Hensel 2017). This shift in thinking posits that the disability mindset makes false dichotomies (“abled, disabled”, “high-functioning, low-functioning”, and “typical, atypical”) that create social biases that are the only meaningful burdens upon neurodivergent individuals, including autistic individuals, from succeeding in the workplace. Furthermore, neurodivergent individuals are no different than neurotypical individuals in the value they derive from work or the rate at which each population wants to work (Ali et al. 2011).

The neurodiversity movement is, however, meaningfully distinct from typical diversity, equity, and inclusion initiatives (Chen et al. 2015; Volkers 2021). As organizations increase their diversity hiring efforts, many organizations assume that inclusion looks the same for neurodiversity as it does for race, gender, age, or sexuality (Bonaccio et al. 2020). Hughes (2020) argued that simply viewing autism through the neurodiversity paradigm may leave one without an awareness of the very real challenges an autistic individual may have, paying mind to the whole spectrum of the diagnosis, at work. Nevertheless, as organizations build out diversity initiatives that at least include disabled workers if not outright focus on them (Hoque et al. 2014; Waterhouse et al. 2010), navigating the balance between the growing social push for inclusion with the, many times, real challenges of accommodating neurodivergent workers across the talent lifecycle is coming into focus for research and applied work.

Inclusion starts with the beginning of the talent lifecycle: attracting candidates to the organization and making hiring decisions about those candidates. A critical component of the talent lifecycle, and of particular interest here, is simply choosing whom to hire. The selection process is a process of intentional barriers—attraction and selection hurdles—meant to attract a qualified candidate pool and winnow down the pool to the best talent. Nevertheless, any stage of the recruiting and selection process may also include unintentional barriers to attracting neurodivergent talent and retaining them through the selection process. Bonaccio et al. (2020) noted that organizations that do not mention disabilities in their diversity initiatives are less likely to attract disabled talent and, even more tactically, job boards themselves may discourage candidates if their designs are not accessible to people with disabilities. The selection procedures discussed throughout the rest of the paper are directly impacted by assumptions made in the recruiting process; an organization that endeavors to include neurodivergent workers into their workforce must first attract them.

Moving into the selection phase of the process, the emphasis changes from not only signaling that the process or tools are inclusive but also that the selection decisions themselves are fair to all candidates. Organizations implement many forms of screening to winnow down their applicant pool. Despite an increasingly virtual and asynchronous world, the most common selection tool likely remains the face-to-face or two-way interview (Campion et al. 1997; Huffcutt et al. 2001). They are so ubiquitous, in fact, that most previous hiring and autism research has focused on how to prepare the autistic candidate for the interview (e.g., Higgins et al. 2008; Kumazaki et al. 2019; Smith et al. 2014; Strickland et al. 2013) with only a recent shift to how the interview can change to be more inclusive (Maras et al. 2021). Growing in parallel to this literature is a growing body of evidence that, put simply, traditional interviews are poorly suited for assessing autistic candidates.

An autistic job candidate may be concerned that their interviewer will form a negative opinion about them for reasons outside of the candidate’s control. The traditional interview is a complex, high-pressure social situation. Past research suggests that managers are uncomfortable interviewing disabled candidates due to a lack of proper training, possible legal implications, or an inability to ask certain questions (Bonaccio et al. 2020). Indeed, Hebl and Skorinko (2005) found that managers react negatively to disability disclosure and efforts have been made to study how to make interviewing less biased against disabled candidates (Reilly et al. 2006). Autistic candidates, meanwhile, are likely to be quite aware of the possible stigma associated with presenting as autistic. Alongside this, research has found that autistic individuals have difficulty understanding non-verbal cues and reciprocal exchanges (Müller 2007), have a harder time processing implied meaning than their neurotypical peers (Wilson and Bishop 2021), and generally experience measurable cognitive disruption when put into social situations (Curioni et al. 2017; Dichter and Belger 2007). Considering both awareness of stigma and possible social-cognitive challenges, it becomes clear that the demands of a traditional job interview set autistic candidates up for failure.

Organizations may consider alternate selection hurdles to be more inclusive or to collect specific job-related information that is difficult to obtain in an interview. Tests, or, more generally, assessments, are typically computer-proctored questions or statements whose content is aligned to the knowledge, skills, abilities, or other characteristics required to perform a job. Often assessments will be built around the job itself (e.g., a work sample, a job simulation, a specific knowledge or skills-based test, etc.) The content of an assessment, however, can vary widely based on the job and organizational needs and constraints. Uniquely job-specific assessments can be expensive and time consuming to develop or impractical (e.g., in the case of entry-level rotational programs), and organizations have opted for assessments of more general job-related traits to circumvent these issues. Personality testing, for example, is frequently used in organizations but many scholars are quick to point out the risks of using personality testing when disabilities are in scope (Hensel 2017; Melson-Silimon et al. 2019) due to the association between some personality traits and mental disabilities.

Cognitive ability testing is another general assessment option available to organizations. Cognitive ability tests assess a candidate’s ability to reason, verbal and mathematical ability, problem-solving skills, memorization, and perceptual and processing speed. Although not without its own problematic history (Hunter and Schmidt 1996; Ployhart and Holtz 2008), cognitive ability has remained an important construct to measure in job selection due to its strong and well-established relationship to job performance (Kuncel et al. 2010; Schmidt and Hunter 1998; Schmidt and Hunter 2004; Schmidt et al. 2016) and is widely used in applied settings today (Bertua et al. 2005; Schmidt and Hunter 2004). The tests usually administered by organizations are typically shorter and narrower in scope than the intelligence tests administered by medical professionals (e.g., Kuo and Eack 2020). Cognitive ability tests used for hiring are designed to estimate an applicant’s potential to use mental processes to solve work-related problems or acquire and apply new job knowledge rather than be sensitive enough to diagnose a disability.

Gamification has emerged as a new format for assessing cognitive ability in response to the conventional long paper-and-pencil-type formats historically used for cognitive-ability assessment. Research has suggested that gamification improves upon traditional formats by offering a more engaging candidate experience and capturing more information via gameplay and trace behaviors (Lumsden et al. 2016; Quiroga et al. 2016). Games have unique features that drive engagement and motivation (Connolly et al. 2012) by providing instantaneous feedback to players (i.e., through level progression, win or loss indicators, and timers; (Burgers et al. 2015; Wood et al. 2004)). Consequently, research has shown that games may reflect true scores better than conventional tests (Miranda and Palmer 2014), games receive more positive reactions and engagement from candidates (Tremblay et al. 2010; Tso et al. 2015), and games may present a challenge that alleviates some of the anxiety associated with traditional tests (Alter et al. 2010; McPherson and Burns 2008). Due to these features, the game-based medium is a promising approach for assessing candidates.

Serious games, or games without an entertainment purpose (Michael and Chen 2006), have been developed from psychometric theory to predict cognitive ability and outcomes related to cognitive ability (Luft et al. 2013; Quiroga et al. 2015). These types of serious games have been found to be strong measures of typical cognitive abilities including spatial reasoning, working memory, and reasoning (Atkins et al. 2014), with a conventional measure of cognitive ability, the Wechsler Adult Intelligence Scales (McPherson and Burns 2008), and with g, or the general mental ability factor (Quiroga et al. 2015). Furthermore, game-based cognitive ability assessments have been shown to be as effective when delivered on a smartphone as on a computer (Brown et al. 2014). Game-based assessments not only provide enhanced candidate experiences but also are similarly effective at measuring cognitive ability as traditional tests.

Games have also been considered in multiple contexts in the autism literature. Most frequently, research has focused on games as either an intervention medium to deliver early treatment or skill development in autistic children (e.g., Bai et al. 2014; Herrera et al. 2008; Malinverni et al. 2017; Murdock et al. 2013; Simut et al. 2016) or, recently, as a way to deliver traditional diagnostic assessments earlier to autistic children, who struggle with the length of these assessments (Mash et al. 2020). Game-like smartphone applications have also been explored as a method for supporting autistic workers by delivering instructions to them in real time (Burke et al. 2010). In short, games appear to be a promising medium for delivering content to autistic individuals in a variety of conditions and applications.

Although cognitive ability is a strong predictor of job performance, the question becomes whether assessing cognitive ability will introduce group differences in passing rates between autistic and neurotypical candidates. In a meta-analysis of cognition and autism spectrum disorder, Velikonja et al. (2019) found that, in general, autistic individuals showed impairments in cognitive functioning relative to neurotypical individuals. These differences were greatest for social cognitive functions, including emotion perception and processing, verbal memory and learning, and processing speed, and the differences were least, and non-significant, for non-social cognitive functions including working memory, attention, and vigilance. Importantly, these are findings based on traditional cognitive batteries and diagnostic settings across the full spectrum of the disorder. As Hughes (2020) noted, there are real medical concerns to consider with this population; however, cognitive impairment varies across the spectrum (Müller 2007) and, within the framework of neurodiversity, removing the social cognitive aspects that unnecessarily and uniquely challenge autistic individuals may attenuate any differences between autistic and neurotypical individuals one could expect from the medical literature. For example, Ozonoff (1995) found that the performance difference between autistic and neurotypical individuals was minimized on a pattern recognition and attention task when it was proctored via a computer instead of an experimenter.

Consequently, the purpose of the present study is to determine whether game-based measures of cognitive ability have potential for assessing job seekers regardless of autism status. Following a review of the medical literature (e.g., Velikonja et al. 2019), one might expect that autistic job seekers will perform worse than their neurotypical peers. However, cognitive ability testing has traditionally been laden with long proctored assessments. Given advancements in technology-proctored delivery modalities (i.e., gamification) that not only remove the need to interact with another person but also make the test-taking experience shorter, less threatening, and capable of being more narrowly focused on non-social cognitive traits, it is predicted that autistic job seekers will have no difficulty performing as well as other job seekers.

**Hypothesis** **1.** *Scores on game-based measures of non-social cognitive ability will not significantly vary across job seekers drawn from an autistic population and the general population.*

The present study is furthermore novel in having the opportunity to study real-world candidates, which comes with its own strengths and limitations. The autistic-candidate data come from a partnership with an organization that helps organizations identify, recruit, and retain professionals (typically college graduates) on the autism spectrum. As part of the application process, candidates are asked to take a hiring assessment which includes game-based assessments. The results are not used to screen out candidates. To match comparison data as closely as possible, general-population applicant scores were sampled from a database of graduate-level job applicants who took the same assessment when applying to similar graduate jobs as the autistic candidates. These general-population candidates are not explicitly neurotypical—hence the use of the “general population” term; it is expected that autistic candidates may be in the general sample in a similar proportion to the base rate of autism in the general population (i.e., approximately 2%; Centers for Disease Control and Prevention 2021).

## 2. Materials and Methods

### 2.1. Power Analysis

Proving a null hypothesis is problematic, and, as the intent of this hypothesis tracks closely to that structure (i.e., two groups will not vary in scores), an a priori step taken was to find what effect size this study could reasonably detect via a power analysis. Velikonja et al. (2019) reported Hedges’ g effect sizes ranged from 0.23 (working memory) to 1.09 (theory of mind), with significant effect sizes beginning at 0.33; interpretation of Hedges’ g can follow the same magnitude interpretation as Cohen’s d. Mean scores on the cognitive ability range from 0 to 5 with a typical standard deviation of 0.6. With sample sizes for each group set to 120, the power analysis indicated that the design would have 80% power to detect a Hedges’ g effect size of 0.36 (approximately a mean difference of 0.22), which is sufficient power to find a significant effect for any significant difference in cognitive ability reported by Velikonja et al. (2019). To reliably find effect sizes as small as 0.23, group sample sizes would need to be approximately 600 with all other parameters remaining equal.

### 2.2. Participants

A total of 586 college-aged participants completed the game-based cognitive ability assessment. A total of 263 candidates were autistic (190 male, 50 female, 23 undisclosed; 179 White, 23 Asian, 23 Hispanic, 11 Black, and 27 undisclosed) and 323 candidates were drawn from the general population (193 male, 107 female, 13 undisclosed; 181 White, 47 Black, 44 Hispanic, 28 Asian, and 13 undisclosed). Sampling from the general population involved searching the assessment database for candidates to entry-level college-graduate jobs who took the same assessment as the autistic candidates. From this group, participants were randomly selected by using a random-number generator to create a roughly equal comparison sample. A detailed demographic breakdown of participants and measures completed is provided in Table 1.

### 2.3. Measures

The games, which are described below, are combined into “packages” of two games, which are taken in sequence. One package, “Disconumbers and Shapedance” combined these two games, and the second, “Digitspan and Shapedance”, switches Disconumbers for Digitspan. Shapedance, which is used in both packages, does not change in any way between the two packages. Scores on the games range from zero to five, with higher scores indicating higher levels of performance on each game. Scores are true interval scores (i.e., values in between whole numbers are possible).

#### 2.3.1. Disconumbers

Disconumbers is a memory and math-based game that asks players to observe a set of numbers on the screen, answer options on the bottom of the screen, and a sequence of highlighted numbers. The purpose of the game is to memorize and tap the sequence of numbers highlighted on the screen in the same order and then calculate the sum of those numbers and select the right option at the bottom of the screen. For example, the screen may show the numbers 1, 6, 3, 8, and 9 in the middle of the screen, 13, 18, 19, and 15 as answer options at the bottom of the screen, and then highlight 1, 8, and 9 in order. Players have a limited time (ten seconds) to tap 1, 8, and 9 in order, correctly add up the number to 18, and select the answer at the bottom. The levels become more challenging as players progress (longer sequences of numbers to memorize, larger numbers, and moving number stimuli in the middle of the screen), and players have a limited time (three minutes) to progress. Losing a level brings a player down to a lower level; only by running out of time does the game end.

#### 2.3.2. Shapedance

Shapedance is a visuospatial ability task similar to the Mental Rotation task (Vandenberg and Kuse 1978) in gameplay. Players are asked to observe a set of matrices, three cells by three cells each, on the screen. Each matrix has an assortment of colored shapes in some of the cells (e.g., a red circle or a blue triangle). In contrast to Mental Rotation tasks, which asks individuals whether two static images would match if rotated, Shapedance asks players to identify the matrices that match one another among several moving matrices. As the player progresses through the game, the number of matches may change, the size of the matrices change, and the matrices may rotate or move on screen, requiring the player to more carefully attend to the stimuli. There is a limited time per level (ten seconds) to correctly identify matches, and players have a limited time (three minutes) to progress as far as possible in the game. Losing a level drops the player down to a lower level. The game only ends when the three minutes have elapsed.

#### 2.3.3. Digitspan

Digitspan is a memorization game very similar to its namesake, the digit span task on the Wechsler Adult Intelligence Scale (Wechsler 1997). The game prompts players with a string of numbers or letters that disappear quickly from the screen. Then, players are asked to use a dial pad on the screen to input the sequence they just saw in a specific manner (e.g., front to back, back to front). The game becomes more challenging as players successfully recall strings; the strings become longer (up to nine digits or letters) and the order in which they must be recalled changes. Similar to the above games, players have a limited time to complete each level (ten seconds) and the entire game (three minutes), losing a level drops one down to a lower level, and the game only ends when time has expired.

### 2.4. Procedure

Two game-based packages were administered across the four samples as part of a longer hiring assessment. After applying to the respective job or program, candidates received an email inviting them to complete the hiring assessment. The broader assessment included, in addition to the games of interest here, five competency-based structured behavioral interview questions that were completed prior to taking the games. At the discretion of the hiring organization, additional unscored questions may have also been included (depending on the organization, these questions are asked to build comfort with the process, inquire about minimum requirements, or ask other job-related questions). Between the interview questions and the games, candidates had an opportunity to take a break (there is no specified limit to the break), so candidates had the opportunity to prepare to take the games.

## 3. Results

**Figure 1:**
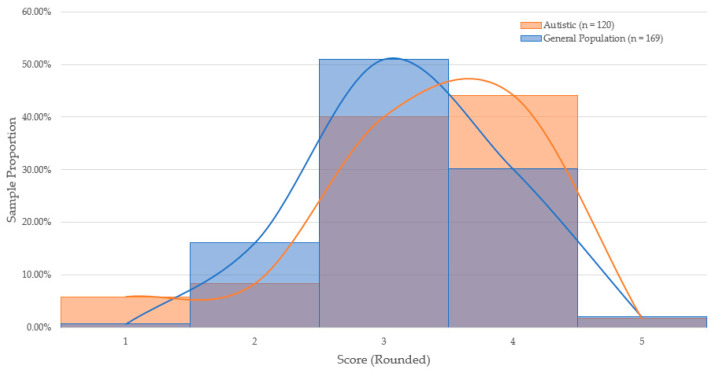
Distribution of Performance on Disconumbers & Shapedance by Sample Proportion.

**Figure 2:**
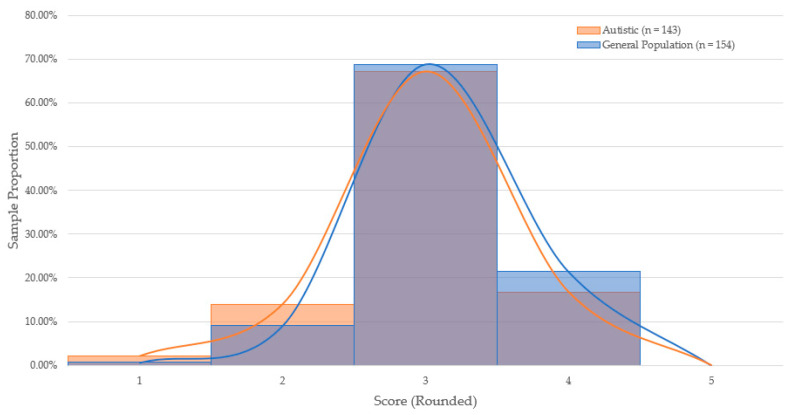
Distribution of Performance on Digitspan & Shapedance by Sample Proportion.

To test whether cognitive ability measurement varied significantly between autistic and general-population candidates, scores obtained in each game-based assessment were compared by group. Two one-way analyses of variance (ANOVA) were conducted to determine whether mean scores on the assessments varied as a function of group membership. Scores on the Disconumbers and Shapedance package did not significantly vary between the general-population (*n* = 169, *M* = 2.81, *SD* = 0.67) and autistic candidates (*n* = 120, *M* = 2.84, *SD* = 0.85): *F*(1,287) = 0.065, *p* = 0.80, and Cohen’s *d* = −0.04. Scores on the Digitspan and Shapedance package varied significantly between the general-population (*n* = 154, *M* = 2.65, *SD* = 0.49) and autistic candidates (*n* = 143, *M* = 2.53, *SD* = 0.58): *F*(1,295) = 3.721, *p* = 0.05, and Cohen’s *d* = 0.22. As shown in Figure 1 and Figure 2, the proportional distribution of scores appear roughly equal across both game packages. The hypothesis of the study was partially supported.

## 4. Discussion

The present study examined whether test scores varied between autistic and general-population graduate job seekers on two game-based cognitive-ability assessments. Three games were used in the two packages: Digitspan, a working memory task similar to the typical used digit span task; Shapedance, a visuospatial ability task that is similar to the Mental Rotation task; and Disconumbers, a working memory and math-based task. Each game is a “serious game” (Michael and Chen 2006), meaning that the intent of the games is not to entertain but rather to leverage game elements to make a functional task more enjoyable and engaging. Game-based measures of cognitive ability were considered in this study for several reasons. First, prior research indicates that cognitive ability is one of the strongest predictors of future job performance (Schmidt et al. 2016), making it a valuable tool in job selection. Second, assessing non-social cognitive traits in a non-social manner may provide a fair way to assess both autistic and neurotypical candidates’ readiness for the same job, and each game measures non-social traits either not typically studied in the literature or found to have the smallest group differences (Velikonja et al. 2019). Although the medical literature reports that cognitive ability is lower in autistic than neurotypical individuals (e.g., Velikonja et al. 2019), other evidence suggests that, in the absence of socially laden cognitive tasks (e.g., being assessed by another person or assessing social cognition, Curioni et al. 2017; Dichter and Belger 2007; Ozonoff 1995), cognition improves in autistic individuals. Third, gamification has been shown to work well in other accommodations and job-related contexts for autistic individuals (e.g., Burke et al. 2010) and in general for increasing candidate engagement and motivation and collecting more data than typical tests (Connolly et al. 2012).

Consistent with the hypothesis of the study, no significant differences or meaningful effect sizes were found between scores on the Disconumbers and Shapedance package between autistic and general population candidates. Inconsistent with the hypothesis of the study but consistent with meta-analytic findings, a significant but small difference was found between autistic and general population candidates’ scores on Digitspan and Shapedance, essentially equivalent to the meta-analytic effect size reported for working memory in Velikonja et al. (2019), which was the smallest effect size found in their study. Although the results are evaluated within the framework of supporting a null hypothesis, it bears noting that the difference between the two groups on the latter game package is small and likely not to introduce problematic group differences at cut scores typically used by organizations (i.e., via empirically set cut scores derived from group passing rates or rationally set cut scores, such as failing the bottom third of candidates). This study’s hypothesis was partially supported: there appears to be a basis to the idea that gamified cognitive-ability assessments can provide a fair means for evaluating autistic candidates.

Studying autistic job seekers is rarely practiced outside of preparing them for job interviews (Higgins et al. 2008; Kumazaki et al. 2019; Smith et al. 2014; Strickland et al. 2013); therefore, this study contributes to the literature by capturing the use of game-based assessments for job selection purposes in an autistic population. More generally, it can be difficult to quantify job-related outcomes for autistic individuals due to a relatively low base rate in the population, a reasonable fear about disclosing a disability status (Bonaccio et al. 2020; Chen et al. 2015), and a reliance on mock procedures meant to prepare autistic candidates, which lack the motivation components of an actual job application, over actual hiring scenarios which are often not feasible. Consequently, the strengths of this study included observing nearly 300 autistic job seekers in a real-world job-application setting. In addition, although game-based technologies have been used both in hiring studies and in autism-specific accommodations and training, this is the first study, to the authors’ knowledge, to explore game-based assessments for selecting autistic job seekers.

As discussed earlier, studies are typically designed to detect a difference or an effect due to some experimental manipulation or relationship to another variable. Although this study was designed in this manner, the study’s hypothesis was a null hypothesis, or a prediction that differences would not exist between groups. Establishing evidence for a lack of a difference is difficult. Simply increasing the sample size increases the power of a test; thus, a statistical test can find even the smallest difference between two groups with sufficient data. In situations such as these, studies look for negligible to small effects sizes coupled with sufficient power to detect meaningful effect sizes in order to rule out accepting the lack of an effect that is due to an underpowered test simply failing to find that effect when it is in fact there (i.e., a type II error). Consequently, the results of this study cannot simply be understood as finding an effect or not, but rather that sufficient data were collected to find meaningful effects. Meaningful effects were not found, as hypothesized, although small effects were detected. Given that studies of this design are unusual and this is the first study to explore this application of game-based cognition assessments to autistic candidates, caution is advised in applying these results without further study and evidence.

Second, the one-way interviews (interviews where the candidate records an answer to a question presented on a screen) that preceded the games may have drained cognitive resources from autistic candidates more so than the general population (Curioni et al. 2017; Dichter and Belger 2007). Given that small group differences were observed for only one package and all candidates could have taken a break between the interviews and games, if desired, it is not expected that the interviews had this effect, although it is a methodological limitation. Future research should consider adding a games-only condition to eliminate the effects any preceding assessment could have on candidates.

Another limitation that should be mentioned is that the current results are not compared to conventional measures of general intelligence. Thus, we cannot demonstrate the extent to which the correspondence in scores between groups is due to the gamification of these tasks or the result of similar standings of actual cognitive ability. However, the gamified tasks in the present research closely resemble non-gamified traditional measures of specific cognitive abilities (Vandenberg and Kuse 1978, Wechsler 1997). Further, we incorporated measures that were shown to exhibit minimal group differences when assessed traditionally, as shown in prior meta-analytic work (Velikonja et al. 2019). Based on this, we suspect similar conclusions would be drawn had a general intelligence measure been evaluated. However, this would be important to confirm via future research.

Lindsay et al. (2019) laid out an empirical supported framework for moving employers from disability discomfort to disability confidence, which involves employers broadening their perspectives through minimizing biases, challenging stigmas, and focusing on the abilities of all candidates. Although nearly ubiquitous in the job-selection process, interviews not only uniquely challenge autistic candidates with non-verbal communication, reciprocal exchanges, and implied meaning (Müller 2007; Wilson and Bishop 2021) but also make hiring managers uncomfortable when the interviewee is disabled (Bonaccio et al. 2020). Ability assessments, when implemented following an alignment between job needs and test content via a job analysis, present an opportunity for organizations to focus on the abilities of their candidates and present unbiased data to hiring managers before they gain an impression of a candidate.

Furthermore, non-traditional assessments can be an opportunity for organizations to signal that they are forward-thinking in their selection process (Bonaccio et al. 2020). Computer-proctored gamified assessments not only possess features that make them more engaging and accessible, but they can also be taken at any place and at any time, making them highly accommodating relative to traditional screening methods, such as interviews. Hence, as the proportion of autistic job seekers only expected to grow (Chen et al. 2015; see also Centers for Disease Control and Prevention 2021), the results of this study suggest that measuring cognition via game-based assessments cannot only fairly assess autistic and neurotypical candidates but may give organizations a head start when hiring in a neurodiverse world.

Nevertheless, caution should be exercised when considering assessments for a neurodiverse population. The games selected for this study measured cognitive traits that aligned with the literature on what traits are least likely to differentiate between autistic and neurotypical individuals (Velikonja et al. 2019). Specifically, these findings do not generalize to other cognitive abilities or other content that can be assessed via games (e.g., emotional intelligence or personality). Others have cautioned against assessing content that has been linked to disabilities—such as emotional intelligence or personality (Hensel 2017; Melson-Silimon et al. 2019)—and although gamification appeared to have helped in this study for a narrow set of cognitive abilities, it is not expected that that pattern will hold with content that autistic individuals typically struggle with, such as social or emotional assessments (Velikonja et al. 2019). For jobs that appear to require social and emotional processing, it may be less a matter of assessment and more a matter of exploring whether accommodations can be made to allow for neurodiverse individuals to productively contribute (e.g., Burke et al. 2010).

The consideration of hiring assessments to promote neurodiversity should also weigh decisions against the risk of adverse impact for other protected classes. Of note, cognitive-ability testing leading to racial differences in hiring rates (i.e., adverse impact) has been so well documented, it has been referred to as a “classic problem” in industrial–organizational psychology (Cottrell et al. 2015, p. 1713; see also Goldstein et al. 2010; Zedeck 2010). Thus, evaluation of the application of the results here should bear this in mind. However, recent research has demonstrated a reason for optimism with regard to racial differences in hiring rates as a function of cognitive-ability testing (Wee et al. 2014), as described further below.

Future research can expand upon this study in several ways. Returning to the discussion of the neurodiversity movement that began the paper, it is important to consider not only the spectrum of autism from a medical perspective (e.g., Hughes 2020), but also who seeks employment from the autistic population in light of the current results. This study’s results are drawn from a subgroup of the autism population, specifically individuals who pursue a university degree. According to Hurley-Hanson and Giannantonio (2016), 35% of autistic individuals pursue a degree. Of them, it is estimated that 85% of autistic college-level job seekers are either unemployed or underemployed, underscoring the need to explore the barriers that prevent this group from successfully attaining employment. That said, the results should be considered within the constraints of this sample: autistic job seekers who are completing or have completed a degree. Future research should consider whether similar interventions (i.e., gamified assessments) are appropriate for non-college-level autistic job seekers where the social barriers of the hiring process are not critical for actually performing the role.

Building from the above discussion of racial differences in hiring due to cognitive-ability testing, recent work has demonstrated that assessment of second-stratum cognitive abilities can be used to select similar quality hires with less risk of adverse impact than general measures of intelligence (Wee et al. 2014). Given that the cognitive assessments packages studied here are specific abilities rather than general intelligence measures, future work should investigate the use of the strategy investigated by Wee et al. (2014) to evaluate whether these game-based assessments can promote equity across multiple protected classes (i.e., both race and mental disability). Moreover, the encouraging results generally found in this study pave the way for exploring other assessment content—i.e., other cognitive abilities or other job-related knowledge, skills, or abilities—as possible improvements to the current selection processes that make finding jobs and persevering through the job-selection process difficult for autistic individuals. Second, gamified assessments may attract neurodiverse candidates to organizations, in line with the signaling effects that Bonaccio et al. (2020) noted that shape candidate perceptions of organizations when searching for jobs. Given a choice between a traditional assessment or a gamified assessment, future research could consider measuring whether the gamified option is more attractive to neurodiverse job seekers. Additionally, future study of gamified assessments, cognitive or otherwise, should evaluate what elements of gamification maintain measurement reliability and validity across neurodiverse job seekers and their neurotypical peers. Lastly, what drives the efficacy of the gamified cognitive-ability assessment remains untested: Future research should explore whether and to what extent the specific cognitive abilities, gamification, and/or on-demand nature of the assessment are responsible for the similar test outcomes between autistic and general-population candidates.

In summary, there are very real barriers that organizations commonly apply to their screening processes that unnecessarily block autistic job seekers from getting jobs that they are otherwise qualified to do. Organizations can make efforts to reduce the unintentional barriers and make fairer decisions grounded in psychometric theory. Game-based assessments of cognitive ability show promise for empowering organizations to make fair, evidence-driven decisions about whom to hire in an increasingly neurodiverse workforce.

## Figures and Tables

**Table 1:** Detailed Demographic Breakdown of Participants and Cognitive Measure Packages.

	Autistic Participant Sample		General Population Sample	
	Disconumbers and Shapedance	Digitspan and Shapedance	Disconumbers and Shapedance	Digitspan and Shapedance
*Total*	120	143	169	154
*Gender*				
Male	81	109	92	101
Female	26	24	67	40
Undisclosed Gender	13	10	0	13
*Race and Ethnicity*				
White	79	100	100	179
Black	5	6	25	22
Hispanic	12	11	22	22
Asian	11	12	12	16
Undisclosed Race/Ethnicity	13	15	0	13

Note: Cognitive assessment packages are denoted by the column heading “Disconumbers and Shapedance” as well as “Digitspan and Shapegance”. Cell values denote samples size in each contingency of demographics and cognitive measure package completed.

## Data Availability

The data presented in this study are available on request from the corresponding author. The data are not publicly available due to privacy concerns.
